# Preclinical Evidence for Antidepressant-like Effects of Histamine H3 Receptor Modulation: A Systematic Review and Meta-Analysis

**DOI:** 10.3390/life16040698

**Published:** 2026-04-21

**Authors:** Ilaria Pullano, Anna Maria Iazzolino, Stefania Landi, Annarita Vignapiano, Francesco Monaco, Luca Steardo

**Affiliations:** 1 Psychiatric Unit, Department of Health Sciences, University Magna Graecia of Catanzaro, 88100 Catanzaro, Italy; pullano.ilaria@gmail.com (I.P.); iazzolinoanna@gmail.com (A.M.I.); 2 Department of Mental Health, Azienda Sanitaria Locale Salerno, 84121 Salerno, Italy; stefanialandi173@gmail.com (S.L.); annarita.vignapiano@gmail.com (A.V.); f.monaco@aslsalerno.it (F.M.); 3 European Biomedical Research Institute of Salerno (EBRIS), 84121 Salerno, Italy

**Keywords:** histamine H3 receptor, antidepressant-like, forced swim test, tail suspension test, sucrose preference, meta-analysis, rodents

## Abstract

Background: Histamine H3 receptor-targeting compounds modulate histaminergic tone and downstream monoaminergic/arousal circuits and have been proposed to exert potential antidepressant-like effects in preclinical models. Methods: We conducted a systematic review and meta-analysis of rodent studies evaluating H3-related interventions on depression-like behavior. We screened 60 records, assessed 12 studies qualitatively (four CORE, eight sensitivity), and included nine studies in random-effects meta-analyses (REML). Primary outcomes were the forced swim test (FST) and tail suspension test (TST); effect sizes were summarized as Hedges’ g (positive values indicate reduced immobility). Results: In the primary ALL analysis, H3-related interventions improved FST outcomes (g = 1.40, 95% CI 0.83–1.97; k = 7) and were also associated with improved TST outcomes, albeit with substantial heterogeneity (g = 2.27, 95% CI 0.80–3.73; k = 5). CORE-only analyses were directionally consistent but less precise (FST: g = 1.11, 95% CI −0.06–2.27; k = 3; TST: g = 2.95, 95% CI 0.87–5.02; k = 2). Sucrose preference was reported in one study and indicated improvement (g = 1.61, 95% CI 0.29–2.92). Conclusions: H3-related interventions show an antidepressant-like signal in rodent FST and TST, with greater heterogeneity for TST, highlighting the need for more standardized and adequately powered preclinical studies.

## 1. Introduction

Major depressive disorder (MDD) remains a leading cause of disability worldwide, and many patients experience incomplete response, delayed onset of benefit, or tolerability limitations with available antidepressants [1]. The recent Global Burden of Disease study estimates confirm its substantial and increasing contribution to the global morbidity of (MDD). Despite the availability of multiple pharmacological treatments, a significant proportion of patients fail to achieve remission, and treatment response is often delayed and incomplete. Large-scale network meta-analyses have highlighted modest differences in efficacy across antidepressants and substantial limitations in tolerability and acceptability, underscoring the need for novel therapeutic targets [2,3,4,5].

These unmet needs motivate the search for novel neurobiological targets beyond classical monoaminergic reuptake mechanisms [6,7,8]. Preclinical research provides a framework to prioritize candidate targets using behavioral paradigms that model specific domains of depressive phenomenology, including behavioral despair and stress-related maladaptation [7,9]. The histaminergic system is increasingly recognized as a modulatory network for mood, cognition, motivation, and sleep–wake regulation. Histamine neurons originating in the tuberomammillary nucleus project widely and interact with neurotransmitter systems implicated in depression, including serotonergic, noradrenergic, dopaminergic, and glutamatergic pathways [10]. The histamine H3 receptor is particularly relevant because it acts predominantly as a presynaptic auto-/heteroreceptor that regulates histamine release and, indirectly, the release of other neurotransmitters [11]. Through this mechanism, H3 receptor-targeting compounds may influence arousal, attention, and affective processing domains frequently disrupted in depression [12,13,14]. In rodents, immobility-based paradigms such as the forced swim test (FST) and tail suspension test (TST) are commonly used for pharmacological screening and are sensitive to many antidepressant treatments [15,16]. However, these assays can be influenced by experimental factors that affect locomotion, arousal, and stress responsivity [17,18]. We cannot exclude that part of the observed effects reflects non-specific changes in arousal or locomotor activity. Because H3-related compounds may alter wakefulness and activity, the interpretation of FST/TST outcomes can vary according to compound, dose/regimen, species/strain, model context (e.g., chronic stress or withdrawal), and scoring procedures [19]. Although species differences (mouse vs. rat) may theoretically influence behavioral outcomes, the quantitative evidence base in the present review was predominantly derived from mouse studies [17]. This methodological diversity contributes to inconsistent findings across the preclinical literature and complicates qualitative interpretation.

Systematic review and meta-analysis offer a structured approach to synthesize heterogeneous evidence, estimate pooled effects, and characterize between-study variability. Although recent reviews have summarized the role of histamine receptors in neuropsychiatric disorders, no previous work has quantitatively synthesized preclinical evidence focusing specifically on H3 receptor modulation and depression-like outcomes. To date, the potential antidepressant-like signal of H3-related interventions in rodent FST/TST outcomes have not been comprehensively summarized with a transparent study-selection workflow and a quantitative synthesis that distinguishes primary analyses from sensitivity checks. Therefore, we conducted a systematic review and meta-analysis of rodent studies evaluating H3-related interventions on depression-like behavior, focusing on FST and TST outcomes, extracting secondary outcomes such as sucrose preference (SPT) when available and performing a CORE-only sensitivity analysis to assess robustness.

## 2. Materials and Methods

### 2.1. Protocol and Reporting Standard

This systematic review was planned a priori and is reported in accordance with the PRISMA 2020 statement [20]. A completed PRISMA 2020 checklist is provided in the Supplementary Materials. The protocol was registered in PROSPERO (CRD420261356601). No substantive amendments to the registered protocol affecting the review question, eligibility criteria, or primary outcomes were made after registration.

### 2.2. Selection Process

Two reviewers (LS and IP) independently screened titles and abstracts and subsequently assessed full-text articles for eligibility against the predefined inclusion and exclusion criteria. Disagreements were resolved by discussion and, when necessary, by consultation with a third reviewer (AMI). Screening decisions, including reasons for exclusion at the full-text stage, were recorded in a standardized database.

### 2.3. Handling of Overlapping and Secondary Publications

When multiple reports appeared to describe overlapping samples, experimental cohorts, or secondary analyses of the same experiment, we planned to include the most complete report and to avoid double-counting data. Potential overlaps were evaluated using study identifiers (authors, year, methods, animal characteristics, intervention details, and outcomes). No eligible duplicate/overlapping datasets contributing to the quantitative synthesis were identified.

### 2.4. Information Sources and Search Strategy

We searched MEDLINE (via PubMed), Embase, and APA PsycINFO from inception to 10 February 2026, which was the date of the last search. Searches combined controlled vocabulary (where available) and free-text keywords for the histamine H3 receptor and H3-targeting compounds together with terms capturing depression-like behavior and relevant behavioral paradigms (e.g., forced swim test and tail suspension test), stress-based models, and anhedonia-related outcomes. Animal-only records were retained by applying an animal/not humans filter, and non-original publication types were excluded (reviews, editorials, comments). The full search strategies for all databases are reported in the Supplementary Materials.

### 2.5. Eligibility Criteria

Studies were eligible if they (1) reported in vivo experiments in rodents; (2) evaluated a H3 receptor-related pharmacological intervention; (3) included an appropriate vehicle/control comparator; and (4) reported at least one eligible depression-relevant behavioral outcome. Primary outcomes were immobility-based behavioral despair paradigms, FST, and/or TST. Secondary outcomes, including anhedonia-related measures such as sucrose preference (SPT), were extracted when available and summarized narratively when insufficient for quantitative synthesis. Studies were excluded if they were not in vivo, not conducted in rodents, lacked a comparator group, evaluated non-eligible interventions, or did not report eligible outcomes. Reports for which the full text could not be retrieved were documented and excluded from the eligibility assessment.

### 2.6. Data Items and Data Extraction

A standardized extraction form was used. Two reviewers independently (I.P. and L.S.J.) extracted data from each included study. Disagreements were resolved through discussion and, when necessary, by consultation with a third reviewer (A.M.I.). No automation tools were used in the data collection process. For each included study we extracted bibliographic information (author, year), animal characteristics (species/strain, sex, age when reported), experimental context/model, intervention details (compound, dose, route, regimen and timing), comparator details, and outcome measures. For the meta-analysis, we extracted group sample sizes, means, and dispersion measures (SD or SEM). These were used to compute Hedges’ g and its variance. When SEM was reported, SD was derived as SEM × √n. Effect sizes were expressed as Hedges’ g (small-sample bias-corrected standardized mean difference) and oriented such that positive values indicate improvement (i.e., reduced immobility in FST/TST). When multiple eligible intervention arms shared a single control group, unit-of-analysis issues were addressed by splitting the control group sample size evenly across comparisons, while retaining the original control mean and standard deviation to avoid double-counting. For each eligible outcome, we sought all reported results that were compatible with the predefined outcome domains. This included all compatible outcome data irrespective of the specific measure format or analysis presentation, whenever sufficient numerical information was available for extraction. When data were missing, unclear, or not numerically extractable, studies were retained in the qualitative synthesis and classified as non-poolable. The reasons are reported in the Supplementary Materials.

### 2.7. Data Synthesis and Statistical Analysis

Studies were grouped for synthesis according to outcome (FST or TST) and according to the predefined analytical sets (ALL and CORE-only sensitivity analyses). Eligibility for each synthesis was determined by matching study characteristics (e.g., intervention type, administration route, and behavioral paradigm) against the predefined inclusion criteria for each dataset.

Effect sizes were synthesized using inverse-variance-weighted random-effects models estimated via restricted maximum likelihood (REML). Separate meta-analyses were conducted for FST and TST, and results are reported as pooled effects with 95% confidence intervals. Between-study heterogeneity was quantified using τ^2^ and I^2^.

Data preparation procedures included conversion of SEM to SD when required and adjustment for shared control groups by splitting the control group sample size across comparisons, as described above.

We prespecified a CORE dataset including study designs deemed most directly comparable across experiments (standard immobility-based paradigms and systemic administration), and a sensitivity dataset including designs with a higher risk of non-comparability (e.g., atypical paradigms, intracranial administration, or contexts likely to amplify arousal/locomotor effects). The CORE dataset was defined a priori to maximize comparability across experiments. Studies were classified as CORE if they met all of the following criteria: (1) systemic administration of the H3-related compound; (2) use of standard immobility-based paradigms (FST or TST); (3) absence of intracranial drug administration; (4) absence of major neurological comorbidity models (e.g., PTZ kindling); and (5) a validated stress-based or genetic depression-like context. Studies not meeting these criteria were retained in the broader sensitivity set. A CORE-only sensitivity analysis was conducted to evaluate robustness of the primary ALL synthesis under stricter study-selection criteria, acknowledging reduced precision due to fewer studies. Given the small number of studies per outcome, formal tests of funnel plot asymmetry were not emphasized, and meta-regression analyses were not performed to avoid model overfitting and unstable moderator estimates.

### 2.8. Risk of Bias Assessment

Risk of bias was assessed at the study level using a structured tool for animal studies (e.g., SYRCLE risk-of-bias tool), covering sequence generation, allocation concealment, blinding, incomplete outcome data, selective reporting, and other sources of bias. Two reviewers performed assessments independently, with disagreements resolved by consensus and third-reviewer adjudication when needed. Study-level risk-of-bias judgements are reported in Table S3.

### 2.9. Assessment of Reporting Bias

Given the small number of studies per outcome (FST: k = 7; TST: k = 5), the formal statistical assessment of reporting bias and small-study effects was considered exploratory and not emphasized, as methods such as funnel plots or Egger’s regression are underpowered when fewer than 10 studies are available.

### 2.10. Certainty of Evidence

The certainty of evidence was not formally assessed using a dedicated framework such as GRADE, because of the exploratory nature of the review, the small number of included preclinical studies, and the marked methodological heterogeneity across animal models and experimental designs.

### 2.11. Software

Analyses were performed in R version 4.3.2 (metafor). Figures were generated from the same analytic pipeline to ensure reproducibility.

## 3. Results

### 3.1. Study Selection

**Figure 1:**
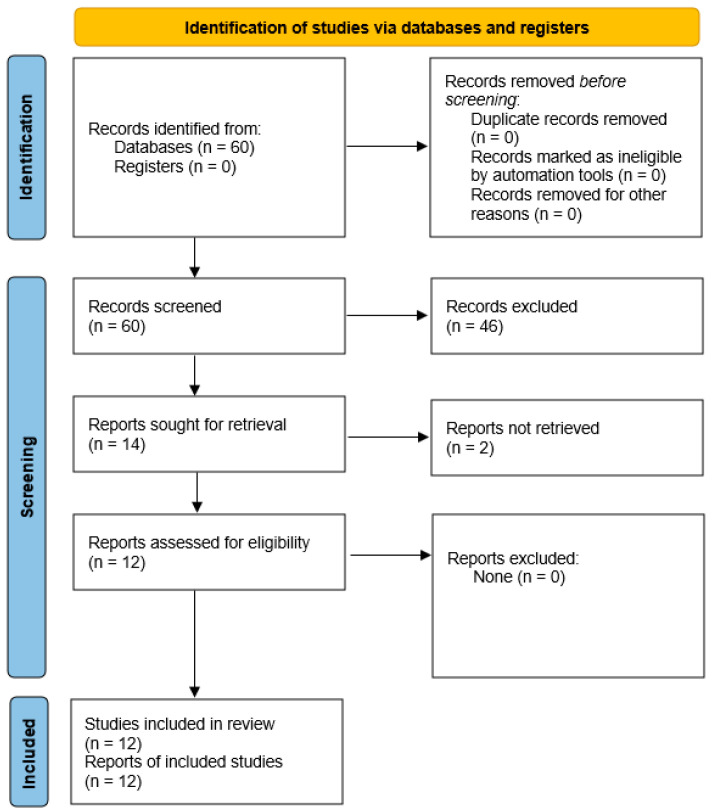
PRISMA 2020 flow diagram summarizing study identification, screening, eligibility assessment, and inclusion.

The search identified 60 records. After duplicate removal (n = 0), 60 records were screened at the title/abstract level and 46 were excluded. Fourteen full-text reports were sought for retrieval, of which two could not be retrieved. Twelve reports were assessed for eligibility, and all met the inclusion criteria; no full-text reports were excluded. Of the 12 included studies, four were CORE studies and eight were sensitivity analyses. Across the poolable comparisons included in the quantitative synthesis, the majority were conducted in mice (all TST comparisons and 6/7 FST comparisons), with only one FST comparison performed in rats (FSL model). Therefore, species-stratified meta-analyses were not feasible. Nine studies provided extractable numeric data and were included in the quantitative synthesis (meta-analysis), yielding seven comparisons for FST and five comparisons for TST (Figure 1).

### 3.2. Included Studies

Twelve studies met the eligibility criteria and were included in the qualitative synthesis (four CORE and eight sensitivity studies; Table 1) [13,21,22,23,24,25,26,27,28,29,30,31]. Across the included studies, rodents were tested in diverse experimental contexts, including stress-related paradigms (e.g., chronic unpredictable stress/corticosterone exposure), genetic vulnerability models, and alcohol withdrawal-related negative affect models. Interventions targeted histamine H3 receptor signaling and included commonly studied H3 antagonists/inverse agonists (e.g., pitolisant, ciproxifan, thioperamide, clobenpropit) as well as H3-relevant compounds (e.g., betahistine, ST-2300), administered via systemic or, in a subset of studies, intracranial routes. Nine studies provided extractable numeric data and were included in the meta-analyses (FST: seven comparisons; TST: five comparisons). Three sensitivity studies were non-poolable due to insufficient numerical reporting (e.g., missing exact group sizes or non-extractable mean/variance estimates) and were summarized qualitatively (Table 1).

### 3.3. Quantitative Synthesis

**Figure 2:**
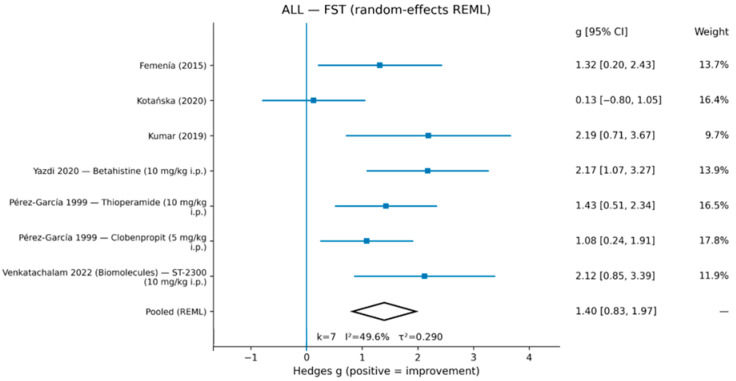
Random-effects meta-analysis (REML) for FST. Effect sizes are Hedges’ g (positive values indicate improvement). Pooled estimate: g = 1.40 [0.83, 1.97], k = 7, I^2^ = 49.6%, τ^2^ = 0.290.

**Figure 3:**
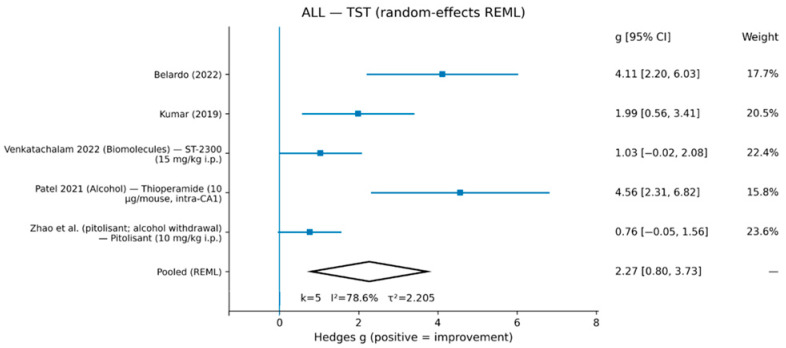
Random-effects meta-analysis (REML) for TST. Effect sizes are Hedges’ g (positive values indicate improvement). Pooled estimate: g = 2.27 [0.80, 3.73], k = 5, I^2^ = 78.6%, τ^2^ = 2.205.

In the primary ALL random-effects REML meta-analysis, H3-related interventions were associated with improved FST outcomes (g = 1.40, 95% CI 0.83–1.97; k = 7), with moderate heterogeneity (I^2^ = 49.6%, τ^2^ = 0.290) (Figure 2; Table 2). For TST, the pooled effect was also in the direction of improvement (g = 2.27, 95% CI 0.80–3.73; k = 5), with substantial heterogeneity (I^2^ = 78.6%, τ^2^ = 2.205) (Figure 3; Table 2). Study-level effect sizes for all comparisons included in the quantitative synthesis are reported in Table 3.

Study-level Hedges’ g (positive values indicate improvement, i.e., reduced immobility) and 95% confidence intervals for each comparison were included in the meta-analyses of the forced swim test (FST; k = 7) and tail suspension test (TST; k = 5). CORE comparisons and sensitivity comparisons were both included in the primary ALL synthesis.

### 3.4. Heterogeneity

Between-study heterogeneity was moderate for FST (I^2^ = 49.6%) and substantial for TST (I^2^ = 78.6%), suggesting that experimental context and design features may influence effect estimates, particularly for TST.

### 3.5. Secondary Outcomes (Anhedonia)

Sucrose preference (SPT) was reported in one included study (Kumar 2019 [13], CUS model) and therefore was not meta-analyzed. In this study, the intervention was associated with higher sucrose preference versus control (Hedges’ g = 1.61, 95% CI 0.29–2.92), consistent with an improvement in anhedonia-like behavior.

### 3.6. Sensitivity Analysis (CORE-Only)

CORE-only sensitivity analyses showed directionally consistent effects with reduced precision due to fewer studies (FST: g = 1.11, 95% CI −0.06–2.27; k = 3; I^2^ = 67.3%, τ^2^ = 0.707; TST: g = 2.95, 95% CI 0.87–5.02; k = 2; I^2^ = 67.2%, τ^2^ = 1.518). While the CORE dataset was defined a priori to improve comparability, we acknowledge that residual heterogeneity remains and that classification may introduce some degree of subjectivity. (Figures S1 and S2).

## 4. Discussion

Through preclinical rodent studies, H3-related interventions were associated with a potential antidepressant-like signal on both FST and TST [12,13,32]. The pooled effect was moderate-to-large for FST with moderate heterogeneity and was larger for TST but with substantial heterogeneity, and included partially overlapping models assessing different aspects of depression-related behavior. This pattern aligns with the neurobiological role of H3 receptors as presynaptic auto-/heteroreceptors that regulate histaminergic tone and modulate downstream neurotransmission relevant to affect and motivation [33,34]. By constraining histamine release and influencing the release probability of other transmitters (notably norepinephrine, dopamine, acetylcholine, and serotonin), H3 signaling can affect arousal, stress responsivity, and cognitive–motivational processes—domains frequently disrupted in depressive states and only partially addressed by conventional antidepressants [11,35,36]. A notable finding is the outcome-specific heterogeneity. Given the substantial methodological heterogeneity, pooled estimates should be interpreted as indicators of a consistent direction of effect rather than precise quantitative estimates of efficacy. FST showed moderate between-study variability, whereas TST displayed substantial heterogeneity, suggesting that TST outcomes may be particularly sensitive to experimental context and design choices [37]. Although species differences are a potential moderator in preclinical behavioral paradigms, the substantial heterogeneity observed for TST cannot be attributed to cross-species variability, as all TST comparisons included in the quantitative synthesis were conducted in mice. For FST, only one comparison was made in rats, limiting the possibility that species differences substantially influenced pooled estimates. Several factors may contribute to this. First, the included studies span diverse models (e.g., stress-based paradigms, genetic vulnerability, neurological comorbidity, and alcohol-withdrawal negative effect), which differ in baseline immobility, stress responsivity, and the neurochemical pathways engaged [38]. Second, intervention characteristics varied (compound identity, dosing regimen, and timing relative to behavioral testing) [39], and a subset used intracranial administration, which can produce effect sizes that are not directly comparable with systemic delivery [40]. Third, immobility-based assays are inherently susceptible to changes in arousal and locomotion [41]. However, locomotor control outcomes were inconsistently reported across studies, limiting our ability to formally evaluate motor/arousal confounding in sensitivity analyses. A systematic evaluation of locomotor control outcomes was not feasible due to inconsistent reporting across studies. This represents a critical limitation, as H3 receptor modulation may influence arousal and activity, potentially leading to false-positive findings in immobility-based paradigms. Because H3 ligands may alter wakefulness, exploratory behavior, and psychomotor activity, apparent “antidepressant-like” effects could be partly driven by non-specific activation or sedation effects in some settings [42]. This is especially relevant for TST, where subtle procedural differences (suspension duration, strain-specific immobility propensity, scoring method, and habituation/handling) can amplify variability across laboratories [43]. It is important to note that FST and TST primarily index immobility-based stress-coping behavior and do not capture the full dimensionality of depressive phenotypes [44]. FST and TST primarily capture behavioral despair and do not encompass core domains of depression such as anhedonia, cognitive dysfunction, or affective bias, limiting their translational validity. Accordingly, the magnitude of pooled effects should be interpreted as domain-specific behavioral signals rather than direct proxies of clinical antidepressant efficacy. Future studies should therefore routinely report locomotor control measures, specify scoring procedures transparently, and ideally preregister primary endpoints to reduce analytic flexibility [45]. From a translational standpoint, the signal observed across paradigms is noteworthy because at least one H3 antagonist (pitolisant) is already clinically approved for sleep–wake indications, supporting the plausibility of drug repurposing pathways. However, the lack of approval in depression highlights a critical translational gap, which may reflect differences in target engagement across disorders, as well as the complexity and heterogeneity of depressive phenotypes compared to sleep–wake regulation processes. Bridging the gap between preclinical findings and clinical efficacy remains challenging, particularly given the complexity of depressive phenotypes and the differences between behavioral paradigms and clinical endpoints [46]. However, the magnitude of pooled effects—particularly for TST—should be interpreted cautiously considering heterogeneity and the limited number of independent studies. Methodological limitations and incomplete reporting in preclinical studies may contribute to effect size inflation, and this should be considered when interpreting the magnitude of pooled effects. Moreover, FST/TST primarily index behavioral despair/immobility and do not capture the full dimensionality of depressive phenotypes [44]. The limited evidence for anhedonia-related outcomes underscores this point: sucrose preference was reported in only one study and suggested improvement, providing preliminary convergence beyond immobility-based paradigms, but insufficient data were available for quantitative pooling. Expanding the outcome portfolio (e.g., anhedonia, social interaction, cognitive/affective bias tasks) and harmonizing reporting would better support translational inference [47].

### 4.1. Limitations

This study has limitations. The small number of included studies per outcome limits the stability of pooled estimates and precludes robust assessment of publication bias or moderator effects. The evidence base is small and methodologically heterogeneous, limiting the feasibility of moderator analyses (including meta-regression) and robust assessment of publication bias. Given the small number of studies per outcome, formal tests of small-study effects are underpowered and were considered exploratory. Reporting quality varied, and key risk-of-bias domains, randomization procedures, allocation concealment, blinding of outcome assessment, and selective reporting were often insufficiently described, which may contribute to effect size inflation in preclinical research. In addition, the certainty of evidence was not formally assessed using a structured framework (e.g., GRADE), which further limits the strength of the conclusions.

Only one rat comparison was included; given the predominance of mouse studies, it is unlikely that species differences substantially influenced pooled estimates. In addition, several studies were non-poolable due to incomplete numeric reporting, which may introduce selection toward studies with extractable results. The small number of rat studies (one poolable comparison) precluded formal moderator analyses by species; thus, future research including balanced cross-species designs would be required to clarify potential species-specific effects. Despite these constraints, consistency in effect direction across the primary ALL synthesis and CORE-only sensitivity analyses supports a detectable antidepressant-like signal for H3-related interventions. Going forward, rigorously designed and adequately powered experiments with ARRIVE-compliant reporting, explicit handling of multi-arm designs, and systematic inclusion of locomotor controls and anhedonia-relevant outcomes will be essential to clarify moderators of response and to define the translational potential of targeting H3 signaling for mood-related symptoms.

### 4.2. Future Directions

Future preclinical studies should (a) adopt standardized FST/TST protocols and report scoring procedures in detail; (b) include locomotor control measures to disentangle antidepressant-like effects from arousal/psychomotor changes; (c) broaden outcome coverage beyond immobility (e.g., sucrose preference and other anhedonia-related endpoints) to improve construct validity; and (iv) use adequately powered, preregistered designs with transparent reporting (ARRIVE-compliant) to enable moderator analyses (e.g., model type, dosing regimen, route of administration, and sex/strain differences). Such improvements will help reduce heterogeneity, strengthen reproducibility, and clarify the translational potential of targeting H3 signaling for mood-related outcomes.

## 5. Conclusions

H3 receptor-related interventions suggest a potential antidepressant-like signal in rodent models, with significant improvements in both FST and TST in random-effects REML meta-analyses. The consistency in effect direction across the primary ALL synthesis and CORE-only sensitivity analyses supports the presence of a detectable signal, although precision decreases when restricted to the smaller CORE subset. Substantial heterogeneity—especially for TST—suggests that experimental context (e.g., stress versus withdrawal paradigms), dosing regimens, route of administration, and procedural differences are likely moderate effect estimates and may contribute to variability across laboratories. Evidence for anhedonia-related outcomes remains limited, with sucrose preference reported in only one study, underscoring the need for broader outcome coverage beyond immobility-based paradigms. Future preclinical studies should prioritize standardized designs and ARRIVE-compliant reporting, include locomotor controls and multiple depression-relevant domains (including anhedonia), and be adequately powered to enable moderator analyses and strengthen translational inference.

## Figures and Tables

**Table 1:** Characteristics of included preclinical studies.

Study	Year	Set	Model/Context	Intervention(s)	Comparator	Outcomes	n (T)	n (C)	Poolable
Belardo et al. [21]	2022	CORE	Social isolation (single-housed)	Single + PEA-OXA (10 mg/kg)	Single + vehicle	TST	7	7	Yes
Femenía et al. [22]	2015	CORE	FSL rat genetic depression model	FSL + clobenpropit 5 mg/kg s.c.	FSL + saline	FST	7	7	Yes
Kotańska et al. [23]	2020	CORE	Chronic corticosterone (20 mg/kg, 21d)	CORT + pitolisant 10 mg/kg	CORT + vehicle (1% Tween)	FST	8	8	Yes
Kumar et al. [13]	2019	CORE	Chronic unpredictable stress (CUS)	CUS + ciproxifan 3 mg/kg/day (CPX-3)	CUS + vehicle	FST, TST, SPT	6	5	Yes
Germundson-Hermanson et al. [24]	2025	Sensitivity	TST time immobile (s) and latency to immobility	H3-related (see study)	Vehicle/control	TST time immobile (s) and latency to immobility	NR	NR	No
Iida et al. [25]	2017	Sensitivity	TST total immobility time (6 min) in LPS-induced depression-like model	H3-related (see study)	Vehicle/control	TST total immobility time (6 min) in LPS-induced depression-like model	NR	NR	No
Lamberti et al. [26]	1998	Sensitivity	FST cumulative immobility (s) for thioperamide, metoprine, L-histidine, H1 agonists; also rota-rod control	H3-related (see study)	Vehicle/control	FST cumulative immobility (s) for thioperamide, metoprine, L-histidine, H1 agonists; also rota-rod control.	NR	NR	No
Patel et al. [27]	2021	Sensitivity	Ethanol withdrawal (24 h) despair; intra-CA1	Thioperamide (10 µg/mouse, intra-CA1)	Vehicle/control	TST	6	6	Yes
Pérez-García et al. [28]	1999	Sensitivity	Acute pharmacological screening (mouse FST)	Thioperamide (10 mg/kg i.p.); Clobenpropit (5 mg/kg i.p.)	Vehicle/control	FST	11–12	11–12	Yes
Venkatachalam et al. [29]	2022	Sensitivity	Acute pharmacological screening (mouse FST); Acute pharmacological screening (mouse TST)	ST-2300 (10 mg/kg i.p.); ST-2300 (15 mg/kg i.p.)	Vehicle/control	FST, TST	6	9	Yes
Yazdi et al. [30]	2020	Sensitivity	PTZ kindling (neurologic comorbidity)	Betahistine (10 mg/kg i.p.)	Vehicle/control	FST	10	10	Yes
Zhao et al. [31]	2026	Sensitivity	IA2BC alcohol withdrawal negative affect (post-EtOH)	Pitolisant (10 mg/kg i.p.)	Vehicle/control	TST	12	12	Yes

Summary of the 12 eligible rodent studies included in the qualitative synthesis (4 CORE and 8 sensitivity studies). Primary outcomes were FST and TST; secondary outcomes (e.g., sucrose preference, SPT) were extracted when available. Studies were classified as poolable when sufficient numeric data were available for effect size computation.

**Table 2:** Summary of meta-analytic results.

Outcome	Analysis	k	Pooled g (95% CI)	I^2^	τ^2^
FST	ALL (primary)	7	1.40 (0.83–1.97)	49.6%	0.290
TST	ALL (primary)	5	2.27 (0.80–3.73)	78.6%	2.205
FST	CORE (sensitivity)	3	1.11 (−0.06–2.27)	67.3%	0.707
TST	CORE (sensitivity)	2	2.95 (0.87–5.02)	67.2%	1.518
SPT	Narrative (single study)	1	1.61 (0.29–2.92)	—	—

Pooled effect estimates from random-effects meta-analyses (REML) for the primary outcomes forced swim test (FST) and tail suspension test (TST), reported as Hedges’ g with 95% confidence intervals (positive values indicate improvement, i.e., reduced immobility). Heterogeneity is summarized using I^2^ and τ^2^. Results are shown for the primary ALL synthesis and for the CORE-only sensitivity analyses. Where applicable, outcomes not suitable for quantitative pooling are indicated as narrative (single study).

**Table 3:** Study-level effect sizes included in the quantitative synthesis (ALL).

Outcome	Set	Study	Model/Context	Intervention	Comparator	n (T/C)	Hedges’ g [95% CI]
FST	CORE	Femenía 2015 [22]	FSL rat genetic depression model	clobenpropit 5 mg/kg s.c.	saline	7/7	1.32 [0.20, 2.43]
FST	CORE	Kotańska 2020 [23]	Chronic corticosterone (20 mg/kg, 21d)	pitolisant 10 mg/kg	vehicle (1% Tween)	8/8	0.13 [−0.80, 1.05]
FST	CORE	Kumar 2019 [13]	Chronic unpredictable stress (CUS)	ciproxifan 3 mg/kg/day	vehicle	6/5	2.19 [0.71, 3.67]
FST	Sensitivity	Yazdi 2020 [30]	PTZ kindling (neurologic comorbidity)	betahistine 10 mg/kg i.p.	control	10/10	2.18 [1.08, 3.28]
FST	Sensitivity	Pérez-García 1999 [28]	Acute screening (mouse FST)	thioperamide 10 mg/kg i.p.	control	11/11	1.43 [0.51, 2.34]
FST	Sensitivity	Pérez-García 1999 [28]	Acute screening (mouse FST)	clobenpropit 5 mg/kg i.p.	control	12/12	1.08 [0.24, 1.91]
FST	Sensitivity	Venkatachalam 2022 [29]	Acute screening (mouse FST)	ST-2300 10 mg/kg i.p.	control	6/9	2.12 [0.85, 3.39]
TST	CORE	Belardo 2022 [21]	Social isolation (single-housed)	PEA-OXA 10 mg/kg	vehicle	7/7	4.11 [2.20, 6.03]
TST	CORE	Kumar 2019 [13]	Chronic unpredictable stress (CUS)	ciproxifan 3 mg/kg/day	vehicle	6/5	1.99 [0.56, 3.41]
TST	Sensitivity	Patel 2021 [27]	Ethanol withdrawal (24 h); intra-CA1	thioperamide 10 µg/mouse (intra-CA1)	control	6/6	4.56 [2.31, 6.82]
TST	Sensitivity	Venkatachalam 2022 [29]	Acute screening (mouse TST)	ST-2300 15 mg/kg i.p.	control	6/9	1.03 [−0.02, 2.08]
TST	Sensitivity	Zhao et al., 2026 [31]	Alcohol withdrawal negative affect (post-EtOH)	pitolisant 10 mg/kg i.p.	control	12/12	0.76 [−0.05, 1.56]

## Data Availability

The data presented in this study are available within the article and its Supplementary Materials. Extracted data and analysis codes may be made available from the corresponding author upon reasonable request.

## Supplementary Materials

The following supporting information can be downloaded at https://www.mdpi.com/article/10.3390/life16040698/s1: Table S1. Study-level effect sizes included in the quantitative synthesis (ALL); Table S2. Non-poolable studies and reasons for exclusion from quantitative synthesis; Table S3. Risk of bias assessment (SYRCLE) across included studies; Figure S1. CORE—FST (random-effects REML). CORE-only sensitivity analysis (random-effects REML) for FST; Figure S2. CORE—TST (random-effects REML). CORE-only sensitivity analysis (random-effects REML) for TST; Figure S3. Leave-one-out analysis for FST (ALL, random-effects REML); Figure S4. Leave-one-out analysis for TST (ALL, random-effects REML); Figure S5: Baujat plot for TST (ALL) and Figure S6. Influence diagnostics for TST (ALL).

## Abbreviations

The following abbreviations are used in this manuscript:

FSL	Flinders Sensitive Line
FST	Forced swim test
H3	Histamine H3 receptor
MDD	Major depressive disorder
REML	Restricted maximum likelihood
SPT	Sucrose preference test
TST	Tail suspension test
