# Supplementary material for: Preclinical Evidence for Antidepressant-like Effects of Histamine H3 Receptor Modulation: A Systematic Review and Meta-Analysis
Source: Life (Basel). 2026 Apr 21;16(4):698. doi: 10.3390/life16040698 (PMC13118249; doi:10.3390/life16040698)
Supplement: Supplementary file 1 [file life-16-00698-s001.zip › life-4266010-supplementary.pdf]

Supplementary Materials

Preclinical Evidence for Antidepressant-Like Effects of Histamine H3 Receptor Modulation: A Systematic Review and Meta-Analysis

Supplementary Methods

Search strategy (MEDLINE via PubMed)

The full search strategies for all databases (MEDLINE via PubMed, Embase, and APA PsycINFO) are reported below. Search was run from inception to 10 February 2026 (date of last search) using the following query (animals-only; excluding reviews/editorials/comments):

MEDLINE (PubMed)

(("Histamine H3 Receptor"[Mesh] OR "H3 histamine receptor"[tiab] OR "histamine H3"[tiab] OR "H3 receptor"[tiab] OR "H3 antagon*"[tiab] OR "H3 inverse agon*"[tiab] OR pitolisant[tiab] OR ciproxifan[tiab] OR thioperamide[tiab]) AND (("forced swim"[tiab] OR FST[tiab] OR Porsolt[tiab]) OR ("tail suspension"[tiab] OR TST[tiab]) OR ("chronic mild stress"[tiab] OR "chronic unpredictable mild stress"[tiab] OR CUMS[tiab] OR CMS[tiab]) OR "learned helplessness"[tiab] OR (anhedoni*[tiab] OR "sucrose preference"[tiab]) OR (depress*[tiab] OR "depression-like"[tiab])) AND (animals[mh] NOT humans[mh])) NOT (review[pt] OR editorial[pt] OR comment[pt])

Embase

((exp histamine h3 receptor/ OR ("H3 histamine receptor" OR "histamine H3" OR "H3 receptor" OR "H3 antagon*" OR "H3 inverse agon*" OR pitolisant OR ciproxifan OR thioperamide).ti,ab,kw.) AND (("forced swim" OR FST OR Porsolt OR "tail suspension" OR TST OR "chronic mild stress" OR "chronic unpredictable mild stress" OR CUMS OR CMS OR "learned helplessness" OR anhedoni* OR "sucrose preference" OR depress* OR "depression-like").ti,ab,kw.) AND (exp animal/ NOT exp human/)) NOT (review/ OR editorial/ OR comment/)

APA PsycINFO

((("H3 histamine receptor" OR "histamine H3" OR "H3 receptor" OR "H3 antagon*" OR "H3 inverse agon*" OR pitolisant OR ciproxifan OR thioperamide).ti,ab,kw.) AND (("forced swim" OR FST OR Porsolt OR "tail suspension" OR TST OR "chronic mild stress" OR "chronic unpredictable mild stress" OR CUMS OR CMS OR "learned helplessness" OR anhedoni* OR "sucrose preference" OR depress* OR "depression-like").ti,ab,kw.) AND (animal.po. OR exp animals/)) NOT (review.pt. OR editorial.pt. OR comment.pt.)

Species/strain was extracted as a predefined data item within the standardized extraction framework. However, because only one poolable rat comparison was available in the quantitative synthesis, species-stratified meta-analyses were not performed.

Supplementary Tables

Table S1. Study-level effect sizes included in the quantitative synthesis (ALL)

**Outcome**	**Set**	**Study**	**Species/Strain**	**Model/Context**	**Intervention**	**Comparator**	**n (T/C)**	**Hedges' g [95% CI]**
FST	CORE	Femenía 2015	Rat (FSL)	FSL rat genetic depression model	FSL + clobenpropit 5 mg/kg s.c.	FSL + saline	7/7	1.32 [0.20, 2.43]
FST	CORE	Kotańska 2020	Mouse (CD-1)	Chronic corticosterone (20 mg/kg, 21d)	CORT + pitolisant 10 mg/kg	CORT + vehicle (1% Tween)	8/8	0.13 [-0.80, 1.05]
FST	CORE	Kumar 2019	Mouse (C57BL/6J)	Chronic Unpredictable Stress (CUS)	CUS + ciproxifan 3 mg/kg/day (CPX-3)	CUS + vehicle	6/5	2.19 [0.71, 3.67]

Outcome	Set	Study	Species/Strain	Model/Context	Intervention	Comparator	n (T/C)	Hedges' g [95% CI]
FST	Sensitivity	Pérez-García 1999	Mouse (OF1)	Acute pharmacological screening (mouse FST)	Thioperamide (10 mg/kg i.p.)	Vehicle/control	11/11	1.43 [0.51, 2.34]
FST	Sensitivity	Pérez-García 1999	Mouse (NR)	Acute pharmacological screening (mouse FST)	Clobenpropit (5 mg/kg i.p.)	Vehicle/control	12/12	1.08 [0.24, 1.91]
FST	Sensitivity	Venkatachalam 2022 (Biomolecules)	Mouse (NR)	Acute pharmacological screening (mouse FST)	ST-2300 (10 mg/kg i.p.)	Vehicle/control	6/9	2.12 [0.85, 3.39]
FST	Sensitivity	Yazdi 2020	Mouse (BALB/c)	PTZ kindling (neurologic comorbidity)	Betahistine (10 mg/kg i.p.)	Vehicle/control	10/10	2.17 [1.07, 3.27]
TST	CORE	Belardo 2022	Mouse (CD1)	Social isolation (single-housed)	Single + PEA-OXA (10 mg/kg)	Single + Vehicle	7/7	4.11 [2.20, 6.03]
TST	CORE	Kumar 2019	Mouse (C57BL/6J)	Chronic Unpredictable Stress (CUS)	CUS + ciproxifan 3 mg/kg/day (CPX-3)	CUS + vehicle	6/5	1.99 [0.56, 3.41]
TST	Sensitivity	Patel 2021 (Alcohol)	Mouse (Swiss albino)	Ethanol withdrawal (24h) despair; intra-CA1	Thioperamide (10 µg/mouse, intra-CA1)	Vehicle/control	6/6	4.56 [2.31, 6.82]

Outcome	Set	Study	Species/Strain	Model/Context	Intervention	Comparator	n (T/C)	Hedges' g [95% CI]
TST	Sensitivity	Venkatachalam 2022 (Biomolecules)	Mouse (C57BL/6)	Acute pharmacological screening (mouse TST)	ST-2300 (15 mg/kg i.p.)	Vehicle/control	6/9	1.03 [-0.02, 2.08]
TST	Sensitivity	Zhao et al. (pitolisant; alcohol withdrawal)	Mouse (C57BL/6J)	IA2BC alcohol withdrawal negative affect (post-EtOH)	Pitolisant (10 mg/kg i.p.)	Vehicle/control	12/12	0.76 [-0.05, 1.56]

Study-level Hedges 'g (positive values indicate improvement, i.e., reduced immobility) and 95% confidence intervals for each comparison included in the meta-analyses of FST and TST.

Table S2. Non-poolable studies and reasons for exclusion from quantitative synthesis

Study	Why non-poolable	What would make it poolable	Outcome(s) present	Notes
Lamberti 1998 (Br J Pharmacol)	Exact n per dose/group not reported (figures state ranges like 10–50 or 11–26); numeric mean±SEM not tabulated; multiple dose-response curves.	Provide exact n and numeric mean±SEM (or SD) for the selected comparison (e.g., thioperamide 5 mg/kg vs saline; cumulative immobility).	FST cumulative immobility (s) for thioperamide, metoprine, L-histidine, H1 agonists; also rota-rod control.	Figures 1–5 show mean±SEM with variable n; best for narrative, or meta only after digitization + exact n per point.

Germundson-Hermanson 2025 (J Neuroimmune Pharmacol)	Sex-stratified group n not reported; figures give only ranges (male n=17–20/group, female n=10–13/group) and scatterplots; mean±SEM not tabulated per subgroup.	Provide exact n + mean±SEM (or SD) for male BLG/Vehicle and male BLG/Thioperamide in TST time-immobile (or supply raw data).	TST time immobile (s) and latency to immobility.	Authors report thioperamide reduced time immobile in male BLG mice vs vehicle (p=0.0050, 2-way ANOVA). Treat as narrative sensitivity unless subgroup n+mean/SEM can be extracted. Approx dot-count from Fig.4b (TST time immobile): Vehicle—Male Sham≈23, Male BLG≈14; Female Sham≈19, Female BLG≈10. Thioperamide—Male Sham≈15, Male BLG≈13; Female Sham≈10, Female

				BLG≈14. Use with caution; exact n still preferred.
Iida 2017 (BBRC) JNJ10181457	TST sample size reported only as a range (n=6–8) for Fig.4C; exact n per group not provided in text/figure caption.	Provide exact n per group (Saline, LPS, JNJ, LPS+JNJ) and numeric mean±SEM/SD for Fig.4C (or raw data).	TST total immobility time (6 min) in LPS-induced depression-like model.	Fig.4C shows JNJ attenuates LPS-induced immobility; data are mean±SEM; groups: Saline, JNJ, LPS, LPS+JNJ.

Studies meeting eligibility criteria but lacking extractable numerical data required for effect-size computation were summarized qualitatively.

Table S3. Risk of bias assessment (SYRCLE) across included studies

**Study**	**Seq. gen**	**Baseline**	**Alloc. conceal**	**Random housing**	**Perf. blind**	**Random outcome assess**	**Detect. blind**	**Incomplete data**	**Selective reporting**	**Other bias**	**Overall**
Belardo 2022	Unclear	Unclear	Unclear	Unclear	**Low**	Unclear	**Low**	Unclear	Unclear	Unclear	Unclear–Moderate
Femenía 2015	Unclear	Unclear	Unclear	Unclear	Unclear	Unclear	**Low**	**Low**	Unclear	Unclear	Unclear–Moderate

Study	Seq. gen	Baseline	Alloc. conceal	Random housing	Perf. blind	Random outcome assess	Detect. blind	Incomplete data	Selective reporting	Other bias	Overall
Kotańska 2020	Unclear	Unclear	Unclear	Unclear	Unclear	Unclear	Low	Low	Unclear	Unclear	Unclear–Moderate
Kumar 2019	Unclear	High	Unclear	Unclear	Unclear	Unclear	Unclear	Unclear	Unclear	High/Unclear	High
Patel 2021 (Alcohol)	Low	Unclear	Unclear	Unclear	Unclear	Unclear	Low	Unclear	Unclear	Low/Unclear	Unclear–Moderate
Pérez-García 1999	Unclear	Unclear	Unclear	Unclear	Unclear	Unclear	Unclear	Low	Unclear	Low/Unclear	Unclear–Moderate
Venkatachalam 2022	Unclear	Unclear	Unclear	Unclear	Low	Unclear	Low	Low	Unclear	Low/Unclear	Unclear–Moderate
Yazdi 2020	Low	Unclear	Unclear	Unclear	Unclear	Unclear	Unclear	Low	Unclear	Unclear	Unclear–Moderate
Zhao et al. 2026	Low	Unclear	Unclear	Unclear	Low	Unclear	Low	Unclear	Unclear	Low/Unclear	Moderate

Study	Seq. gen	Baseline	Alloc. conceal	Random housing	Perf. blind	Random outcome assess	Detect. blind	Incomplete data	Selective reporting	Other bias	Overall
Lamberti 1998 *(non-poolable)*	Unclear	Unclear	Unclear	Unclear	Unclear	Unclear	Unclear	Unclear	Unclear	Low/Unclear	Unclear–Moderate
Germundson-Hermanson 2025 *(non-poolable)*	Low	Unclear	Unclear	Unclear	Unclear	Unclear	Low	Unclear	Unclear	Unclear	Unclear–Moderate
Iida 2017 *(non-poolable)*	Unclear	Unclear	Unclear	Unclear	Unclear	Unclear	Unclear	Unclear	Unclear	Unclear	Unclear–Moderate

Risk of bias was assessed at the study level using SYRCLE domains for animal intervention studies. Each domain was judged as Low risk, High risk, or Unclear risk based on reporting and methods. Disagreements were resolved by consensus.

Supplementary Figures

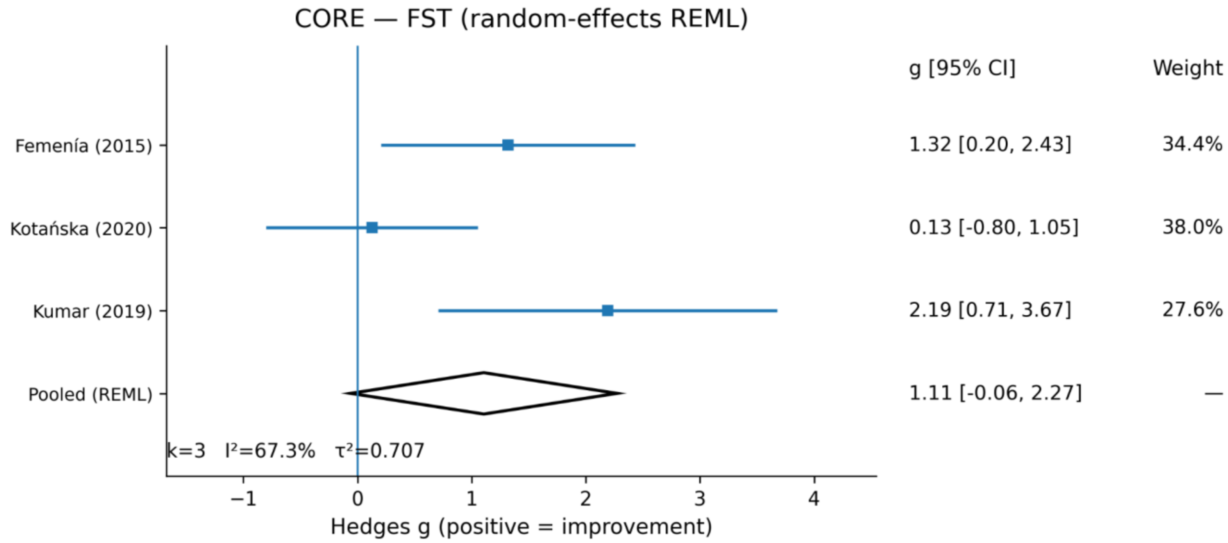

Figure S1. CORE — FST (random-effects REML). CORE-only sensitivity analysis (random-effects REML) for FST. Pooled estimate: g=1.11 [−0.06, 2.27], k=3, I^2=67.3%, τ^2=0.707.

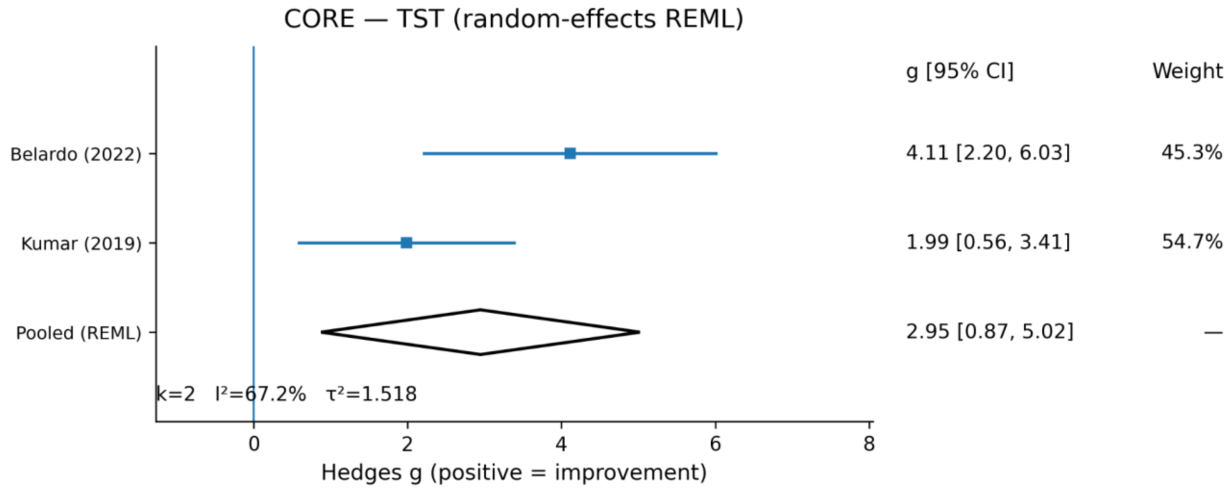

Figure S2. CORE — TST (random-effects REML). CORE-only sensitivity analysis (random-effects REML) for TST. Pooled estimate: g=2.95 [0.87, 5.02], k=2, I^2=67.2%, τ^2=1.518.

Influence and Sensitivity Analyses

To assess robustness of the primary ALL syntheses, we conducted leave-one-out analyses and influence diagnostics. For TST (k=5), we additionally examined study contributions to heterogeneity using a Baujat plot and repeated the meta-analysis excluding the intracranial administration study (Patel 2021) as a design-based sensitivity check. Leave-one-out analyses showed that removal of any single study did not reverse the direction of the pooled effect. Excluding the intracranial administration study (Patel 2021) reduced the pooled TST estimate to g=1.78 (95% CI 0.45 to 3.11) and decreased heterogeneity (τ^2=1.391; I^2=73.1%).

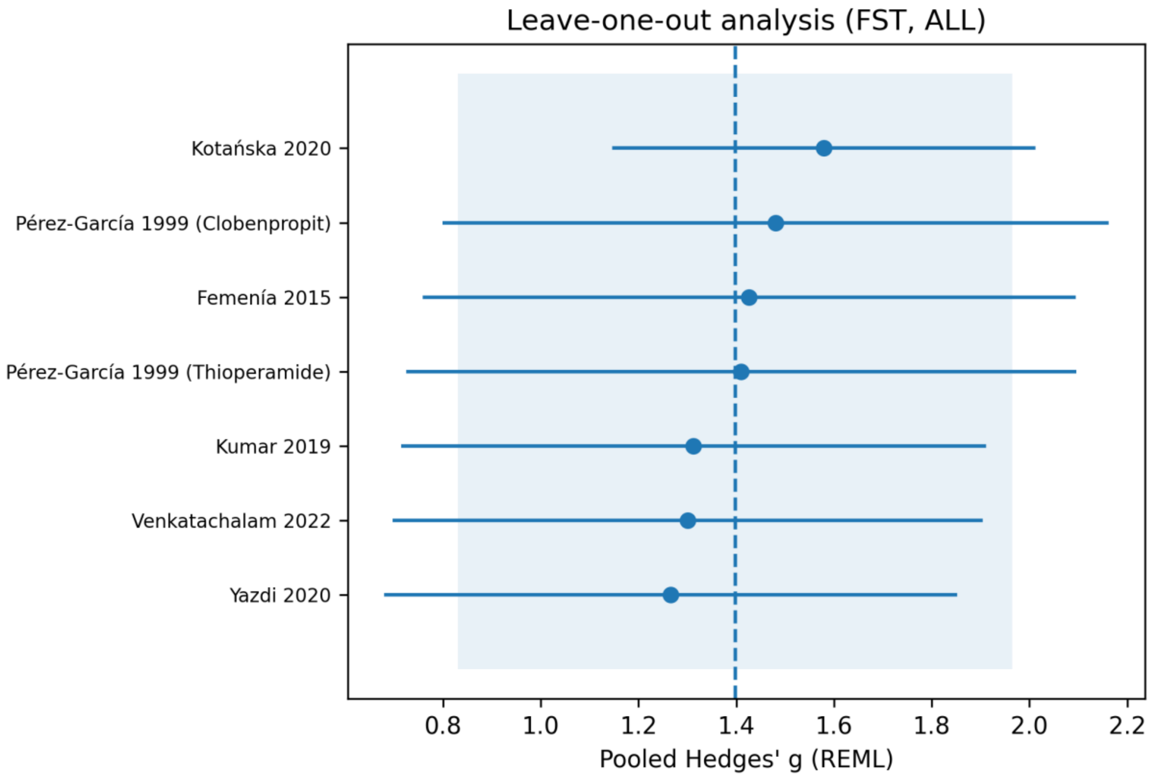

Figure S3. Leave-one-out analysis for FST (ALL, random-effects REML). Each point shows the pooled estimate after removing the indicated study; dashed line indicates the overall pooled effect and shaded band its 95% CI.

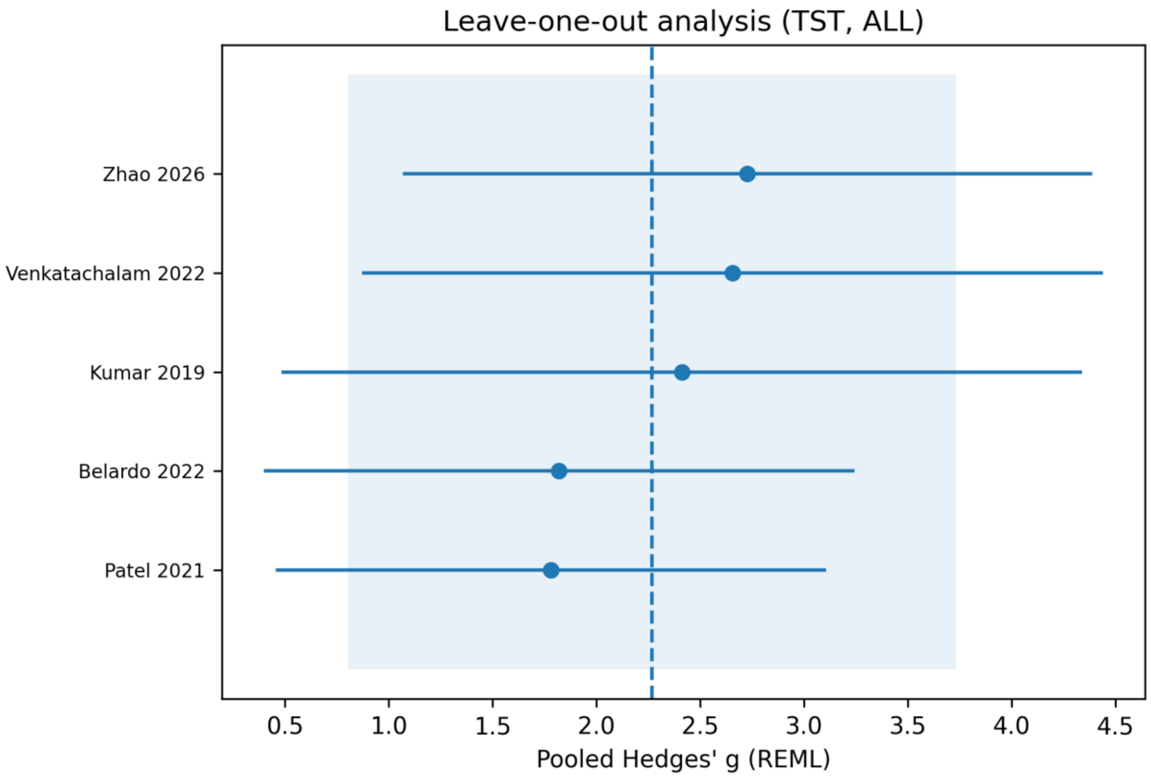

Figure S4. Leave-one-out analysis for TST (ALL, random-effects REML). Each point shows the pooled estimate after removing the indicated study; dashed line indicates the overall pooled effect and shaded band its 95% CI.

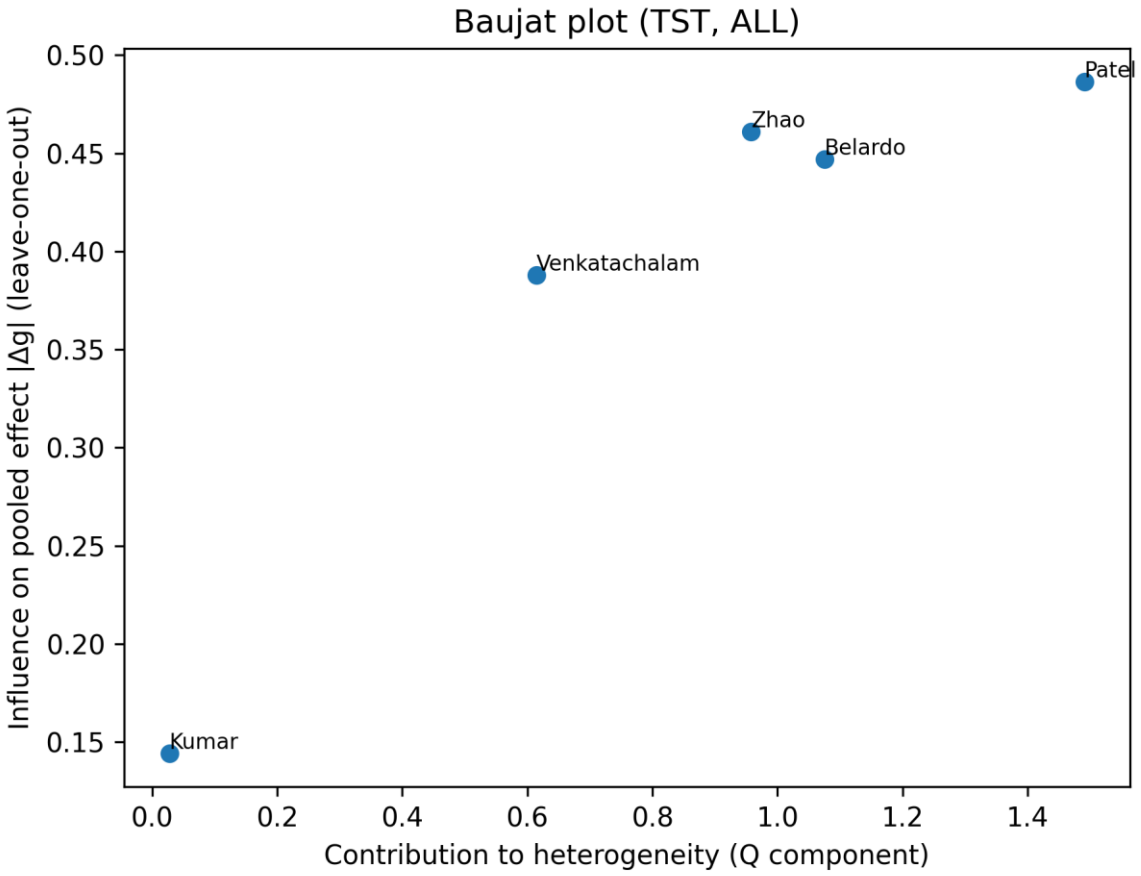

Figure S5. Baujat plot for TST (ALL). X-axis: contribution to heterogeneity (Q component) under the random-effects model; Y-axis: influence on pooled effect (absolute change in pooled g under leave-one-out).

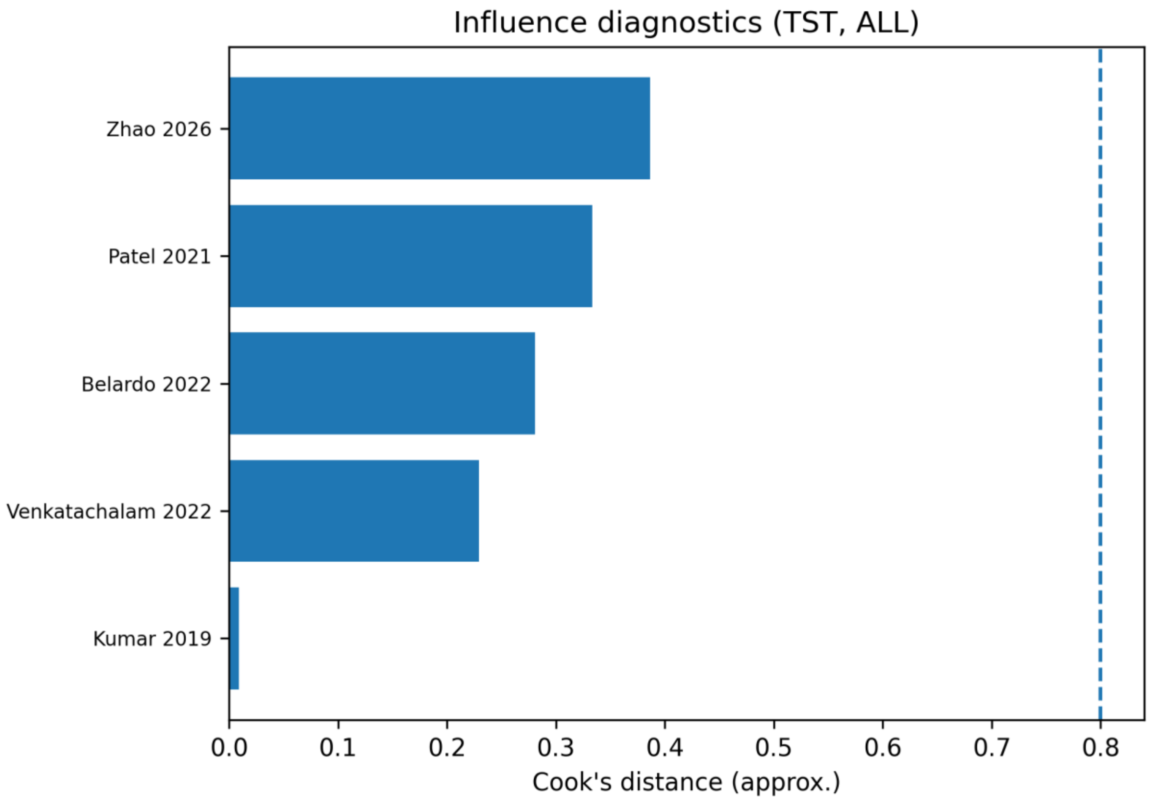

Figure S6. Influence diagnostics for TST (ALL). Approximate Cook's distance values highlight comparisons with larger influence on the pooled estimate (dashed line indicates 4/k heuristic).

PRISMA 2020 Checklist

Section and Topic	Item #	Checklist item	Location where item is reported
**TITLE**			
Title	1	Identify the report as a systematic review.	Title page
**ABSTRACT**			
Abstract	2	See the PRISMA 2020 for Abstracts checklist.	Abstract section
**INTRODUCTION**			
Rationale	3	Describe the rationale for the review in the context of existing knowledge.	Introduction
Objectives	4	Provide an explicit statement of the objective(s) or question(s) the review addresses.	End of Introduction
**METHODS**			
Eligibility criteria	5	Specify the inclusion and exclusion criteria for the review and how studies were grouped for the syntheses.	Section 2.5
Information sources	6	Specify all databases, registers, websites, organisations, reference lists and other sources searched or consulted to identify studies. Specify the date when each source was last searched or consulted.	Section 2.4

Section and Topic	Item #	Checklist item	Location where item is reported
Search strategy	7	Present the full search strategies for all databases, registers and websites, including any filters and limits used.	Supplementary Materials
Selection process	8	Specify the methods used to decide whether a study met the inclusion criteria of the review, including how many reviewers screened each record and each report retrieved, whether they worked independently, and if applicable, details of automation tools used in the process.	Section 2.2
Data collection process	9	Specify the methods used to collect data from reports, including how many reviewers collected data from each report, whether they worked independently, any processes for obtaining or confirming data from study investigators, and if applicable, details of automation tools used in the process.	Section 2.6
Data items	10a	List and define all outcomes for which data were sought. Specify whether all results that were compatible with each outcome domain in each study were sought (e.g. for all measures, time points, analyses), and if not, the methods used to decide which results to collect.	Section 2.5 and 2.6
	10b	List and define all other variables for which data were sought (e.g. participant and intervention characteristics, funding sources). Describe any assumptions made about any missing or unclear information.	Section 2.6
Study risk of bias assessment	11	Specify the methods used to assess risk of bias in the included studies, including details of the tool(s) used, how many reviewers assessed each study and whether they worked independently, and if applicable, details of automation tools used in the process.	Section 2.8 and Supplementary Materials Table S3
Effect measures	12	Specify for each outcome the effect measure(s) (e.g. risk ratio, mean difference) used in the synthesis or presentation of results.	Section 2.6
Synthesis methods	13a	Describe the processes used to decide which studies were eligible for each synthesis (e.g. tabulating the study intervention characteristics and comparing against the planned groups for each synthesis (item #5)).	Section 2.7
	13b	Describe any methods required to prepare the data for presentation or synthesis, such as handling of missing summary statistics, or data conversions.	Section 2.6 and 2.7
	13c	Describe any methods used to tabulate or visually display results of individual studies and syntheses.	Section 2.7; Figures 2-3; Tables 2-3
	13d	Describe any methods used to synthesize results and provide a rationale for the choice(s). If meta-analysis was performed, describe the model(s), method(s) to identify the presence and extent of statistical heterogeneity, and software package(s) used.	Section 2.7
	13e	Describe any methods used to explore possible causes of heterogeneity among study results (e.g. subgroup analysis, meta-regression).	Section 2.7
	13f	Describe any sensitivity analyses conducted to assess robustness of the synthesized results.	Section 2.7
Reporting bias assessment	14	Describe any methods used to assess risk of bias due to missing results in a synthesis (arising from reporting biases).	Section 2.9
Certainty assessment	15	Describe any methods used to assess certainty (or confidence) in the body of evidence for an outcome.	Section 2.10
**RESULTS**			
Study selection	16a	Describe the results of the search and selection process, from the number of records identified in the search to the number of studies included in the review, ideally using a flow diagram.	Section 3.1; Figure 1

Section and Topic	Item #	Checklist item	Location where item is reported
	16b	Cite studies that might appear to meet the inclusion criteria, but which were excluded, and explain why they were excluded.	Section 3.1; Supplementary Materials Table S2
Study characteristics	17	Cite each included study and present its characteristics.	Section 3.2; Table 1
Risk of bias in studies	18	Present assessments of risk of bias for each included study.	Section 2.8; Supplementary Materials Table S3
Results of individual studies	19	For all outcomes, present, for each study: (a) summary statistics for each group (where appropriate) and (b) an effect estimate and its precision (e.g. confidence/credible interval), ideally using structured tables or plots.	Section 3.3; Table 3
Results of syntheses	20a	For each synthesis, briefly summarise the characteristics and risk of bias among contributing studies.	Section 3.2
	20b	Present results of all statistical syntheses conducted. If meta-analysis was done, present for each the summary estimate and its precision (e.g. confidence/credible interval) and measures of statistical heterogeneity. If comparing groups, describe the direction of the effect.	Section 3.3; Figures 2-3; Table 2
	20c	Present results of all investigations of possible causes of heterogeneity among study results.	Section 3.4; Section 4
	20d	Present results of all sensitivity analyses conducted to assess the robustness of the synthesized results.	Section 3.6; Supplementary Materials Figure S1-S2
Reporting biases	21	Present assessments of risk of bias due to missing results (arising from reporting biases) for each synthesis assessed.	Section 2.9
Certainty of evidence	22	Present assessments of certainty (or confidence) in the body of evidence for each outcome assessed.	Section 2.10; Section 4.1
**DISCUSSION**			
Discussion	23a	Provide a general interpretation of the results in the context of other evidence.	Section 4
	23b	Discuss any limitations of the evidence included in the review.	Section 4.1
	23c	Discuss any limitations of the review processes used.	Section 4.1
	23d	Discuss implications of the results for practice, policy, and future research.	Section 4.2
**OTHER INFORMATION**			
Registration and protocol	24a	Provide registration information for the review, including register name and registration number, or state that the review was not registered.	Section 2.1
	24b	Indicate where the review protocol can be accessed, or state that a protocol was not prepared.	Section 2.1
	24c	Describe and explain any amendments to information provided at registration or in the protocol.	Section 2.1
Support	25	Describe sources of financial or non-financial support for the review, and the role of the funders or sponsors in the review.	Funding section
Competing interests	26	Declare any competing interests of review authors.	Conflicts of Interest section
Availability of	27	Report which of the following are publicly available and where they can	Data

Section and Topic	Item #	Checklist item	Location where item is reported
data, code and other materials		be found: template data collection forms; data extracted from included studies; data used for all analyses; analytic code; any other materials used in the review.	Availability Statement; Supplementary Materials
